# First Insights into the Gut Microbiota of Mexican Patients with Celiac Disease and Non-Celiac Gluten Sensitivity

**DOI:** 10.3390/nu10111641

**Published:** 2018-11-02

**Authors:** Jose F. Garcia-Mazcorro, Xaira Rivera-Gutierrez, Orestes De Jesus Cobos-Quevedo, Peter Grube-Pagola, Arturo Meixueiro-Daza, Karina Hernandez-Flores, Francisco J. Cabrera-Jorge, Hector Vivanco-Cid, Scot E. Dowd, Jose M. Remes-Troche

**Affiliations:** 1 Instituto de Investigaciones Medico Biológicas, Universidad Veracruzana, Calle Agustín de Iturbide, Salvador Díaz Mirón, 91700 Veracruz, México; josegarcia_mex@hotmail.com (J.F.G.-M.); xaai.rg@gmail.com (X.R.-G.); orestes.cq7@gmail.com (O.D.J.C.-Q.); grubejr78@gmail.com (P.G.-P.); arturomeixueiro@hotmail.com (A.M.-D.); karinahernandezflores@gmail.com (K.H.-F.); fco-javier-cj@hotmail.com (F.J.C.-J.); hvivanco@uv.mx (H.V.-C.); 2 Molecular Research LP, 503 Clovis Rd, Shallowater, TX 79363, USA; sdowd@mrdnalab.com; 3 Laboratorio de Fisiología Digestiva y Motilidad Gastrointestinal, Instituto de Investigaciones Medico Biológicas, Universidad Veracruzana, Calle Agustín de Iturbide, Salvador Díaz Mirón, 91700 Veracruz, México

**Keywords:** gluten-related disorders, celiac disease, gut microbiota, gluten-free diet, *Pseudomonas*

## Abstract

Gluten-related disorders (GRDs) are common chronic enteropathies and increasing evidence suggests an involvement of the gut microbiota. We examined the gut microbiota in Mexican people afflicted with GRDs. Ultra-high-throughput 16S marker sequencing was used to deeply describe the duodenal and fecal microbiota of patients with celiac disease (CD, *n* = 6), non-celiac gluten sensitivity (NCGS, *n* = 12), and healthy subjects (*n* = 12) from our local area. Additionally, we also investigated the changes in gut microbiota after four weeks on a gluten-free diet (GFD) in a subset of patients from whom paired samples were available. Despite a high inter-individual variability, significant differences in various microbial populations were identified. The linear discriminant analysis (LDA) effect size (LEfSe) method revealed that the genus *Actinobacillus* and the family Ruminococcaceae were higher in the duodenal and fecal microbiota of NCGS patients, respectively, while *Novispirillum* was higher in the duodenum of CD patients (*p* < 0.05, LDA score > 3.5). Interestingly, paired samples from NCGS patients showed a significant difference in duodenal *Pseudomonas* between the baseline period (median: 1.3%; min/max: 0.47–6.8%) and the period after four weeks on GFD (14.8%; 2.3–38.5%, *p* < 0.01, Wilcoxon signed-rank test). These results encourage more research on GRDs in México.

## 1. Introduction

The gut microbiota is comprised of thousands of microbial species that vary widely among individuals [1] and also over time within the same individual due to environmental factors such as dietary patterns [2]. The gut microbiota helps modulate the immune system [3] and has been associated with diseases related to the alimentary tract such as obesity and inflammatory bowel diseases [4]. Given the close relationship between the immune system and microorganisms inside the gut, it is believed that most disorders of the digestive tract bear some relationship with the gut microbiota although a cause-effect relationship can hardly be established [5]. 

Gluten-related disorder (GRD) is a general term to describe all maladies triggered by gluten, with celiac disease (CD) being the most studied. CD is an autoimmune disorder where the consumption of gluten leads to an abnormal T cell-mediated immune response and damage to epithelial cells in genetically susceptible individuals [6,7]. Other factors related to CD include perinatal environmental factors such as the duration of breastfeeding as well as gut-microbiota interactions [8] and the only available treatment for GRDs is a life-long consumption of a gluten-free diet (GFD). On the other hand, non-celiac gluten sensitivity (NCGS) is a different GRD yet it also responds to a gluten-free diet (GFD) [9,10]. The diagnosis of NCGS is based on the clinical response to GFD and the exclusion of other syndromes as there is no NCGS-specific biomarker yet identified like in wheat’s allergy (e.g., the presence of IgE) or CD (e.g., the presence of TG2 antibodies) [11]. 

Growing evidence suggests that the gut microbiota is closely related to GRDs, particularly CD [8,12]; however, a disease-specific microbial signature of GRDs has not yet been defined and there is a lack of consensus with respect to the specific changes involved in these disorders with or without dietary gluten [12,13,14,15], partly due to the well-known high interindividual variation of the gut microbiota [16,17]. One study used culture techniques to investigate the effect of GFD on fecal *Bifidobacterium* and showed that CD patients have a lower load of this microorganism [18]. However, it is more informative to analyze all (or most) members of the gut microbiota to reach biologically feasible and clinically useful conclusions. In this regard, several studies have used massive high-throughput sequencing technologies to do so but have mostly focused on child populations [19,20]. Another study analyzed the fecal microbiota in 21 adults from the Netherlands before, during and after four weeks on GFD but did so in healthy control volunteers only [21]. Interestingly, the authors showed that a decreased abundance of Veillonellaceae was a distinctive feature during the consumption of GFD [21]. 

In México, CD has a prevalence of ~1% (~1.2 million people) [22], yet we know very little about CD in terms of its genetic predisposition, clinical presentation, treatment and involvement of the gut microbiota in Mexican patients [17,23,24,25,26,27]. The purpose of this research is to investigate the gut microbiota composition and predicted functional profile in Mexican patients with GRDs. To our knowledge, this work represents the first effort to investigate the gut microbiota in these important clinical conditions in México. Additionally, we also investigated the changes in the gut microbiota after four weeks on a gluten-free diet (GFD) in a subset of patients from whom paired samples were available. 

## 2. Materials and Methods

### 2.1. Ethical Considerations

This study was conceived with the combined knowledge and expertise of clinical and biomedical scientists from the Instituto de Investigaciones Medico Biologicas at the Universidad Veracruzana. Informed consent was obtained from all subjects and the study was approved by the local ethics committee (IIMB-UV 2016/011). 

### 2.2. Recruitment of Participants

Consecutive newly diagnosed CD and NCGS subjects were recruited and evaluated over six months from patients attending the Department of Gastroenterology of the Universidad Veracruzana in Veracruz, México. CD diagnosis was based on the presence of CD-specific antibodies, genetic markers and histological examination; NCGS diagnosis was made during the patient’s consultation if subjects had symptoms related to the ingestion of gluten (e.g., bloating, flatulence, altered bowel habits, and muscle pains) but no CD-specific antibodies and negative biopsies at the baseline (see “2.1 Subject enrollment” in Supplementary Information for more detailed explanations). Healthy volunteers with no history of digestive pathologies, lack of CD-specific antibodies and normal biopsies at baseline, were also included in the study. Blood samples, small bowel (i.e., proximal duodenum) mucosal biopsies, and fecal samples were obtained from the majority of the subjects although many patients refused to provide stool samples. As mentioned before, we additionally sought to investigate the potential microbial signatures associated with the consumption of certified gluten-free foods, where adherence to the GFD was defined if the subjects kept the diet >90% of the recorded time using diary records (see “2.2 GFD intervention” in Supplementary Information). 

### 2.3. DNA Extraction, PCR, and 16S rDNA Sequencing

Biopsy and fecal samples were used to obtain the total genomic DNA samples for further PCR and sequencing of the 16S rRNA gene (16S rDNA) as shown elsewhere [28,29]. Briefly, we used a bead-beating coupled with a commercial DNA extraction kit (Wizard^®^ Genomic DNA Purification, PROMEGA, Madison, WI, USA) and samples were normalized to 100 ng/uL for further analysis. We used primers 515F (GTGYCAGCMGCCGCGGTAA) and 806R (GGACTACNVGGGTWTCTAAT) to amplify the V4 region of the 16S rDNA as suggested by the Earth Microbiome Project. Purified PCR products were used to prepare the DNA libraries using the Illumina TruSeq DNA library preparation protocol. Sequencing was performed in a MiSeq instrument (Illumina) at Molecular Research LP (MR DNA, Shallowater, TX, USA) following the manufacturer’s instructions. 

### 2.4. Bioinformatics

The open-source bioinformatics pipeline Quantitative Insights into Microbial Ecology [30] v.1.8 was used for most of the core analyses. Operational Taxonomic Units (OTUs) were chosen using two approaches. First, using the pick_open_reference_otus.py accordingly to the suggestions by Rideout et al. [31]. This approach is capable of detecting OTUs that are not necessarily represented in the reference databases. Further taxonomic and diversity analyses were performed using all OTUs (i.e., the full OTU table) and a filtered OTU table (OTUs with <0.005% of all sequences were removed as suggested by Navas et al. [32]. Second, using the pick_closed_reference_otus.py to then be able to use the OTU table for the prediction of functional metagenome using Phylogenetic Investigation of Communities by Reconstruction of Unobserved States (PICRUSt) [33]. The GreenGenes database [34] at 97% similarity was used as the reference 16S database. All sequence and metadata information are publicly available (NCBI, PRJNA401920).

### 2.5. Statistical Analysis

A chi-squared test was used to compare the frequencies (e.g., the proportion of women, number of patients showing a clinical improvement) and the non-parametric Kruskal–Wallis test was used for comparison of health parameters (e.g., blood parameters) and microbial groups. The linear discriminant analysis (LDA) effect size (LEfSe, [35]) was used to determine the organisms that explain the differences in microbiota. Please note that in LEfSe, the idea is that the significant biomarkers (in this case microbial phylogroups) are ranked based on the effect size (the magnitude of the variation) rather than on the statistical significance. When comparing two sets of data (e.g., before and after GFD), the Wilcoxon signed-rank test or the Mann–Whitney test were used. The unique fraction metric (UniFrac) was used to measure the phylogenetic distance among taxa [36]. Both weighted and unweighted UniFrac were calculated and analyzed using Principal Coordinate Analyses (PCoA) because they can lead to different insights into the factors that shape the composition of bacterial communities [37,38]. The ANOSIM and Adonis tests were used to determine whether the grouping of samples by a given category (e.g., health status) is statistically significant based on the UniFrac distances. Two age groups (young < 35 years; old > 35 years) and two body mass index (BMI) groups (low < 24.5; high > 24.5) were created to evaluate the potential contribution of these factors to the similarity of bacterial communities. STAMP [39] was used to analyze PICRUSt data using non-parametric tests. 

## 3. Results

### 3.1. Subjects

A total of six patients with CD, twelve patients with NCGS and twelve control subjects were successfully enrolled over the six months enrollment period (Table 1). Please note that not all samples were obtained from all subjects mainly because of the lack of compliance, especially with the submission of stool samples. Among the CD patients, one had a Marsh I classification, two had Marsh II and three had Marsh IIIa. The impact of these varying baseline scores on clinical development and gut microbiota is uncertain but something to look for in future studies. The history of CD among relatives was more common in CD patients, CD patients had lower BMIs and hemoglobin levels and higher intraepithelial lymphocyte counts (Table 2). There was no difference between the CD and NCGS patients at baseline with regards to abdominal pain and bloating (Table 2). 

### 3.2. 16S Sequencing and Taxonomic Classification of Sequence Reads

A total of 2.3 million (biopsies, *n* = 30) and 1.5 million (fecal, *n* = 14, many patients refused to provide a stool sample) good-quality 16S rDNA sequences (median length: 300 base pairs) were obtained from the baseline samples and used for OTU picking and further analyses. A total of 32,800 OTUs were originally detected using the open OTU picking approach (unfiltered OTU table); the removal of low-abundant OTUs (i.e., OTUs with <0.005% of total reads) yielded 975 and 916 OTUs (only ~3% of all original OTUs) in biopsy and fecal samples, respectively. It is outside the scope of this current work to discuss the consequences of removing low abundant OTUs but please be aware that the so-called rare microbes may in fact be keystone species regulating the function of different microbial environments, including host-associated microbiomes [40]. 

### 3.3. Microbiota at Baseline

#### 3.3.1. Microbiota in Duodenal Biopsy Samples at Baseline

**Figure 1:**
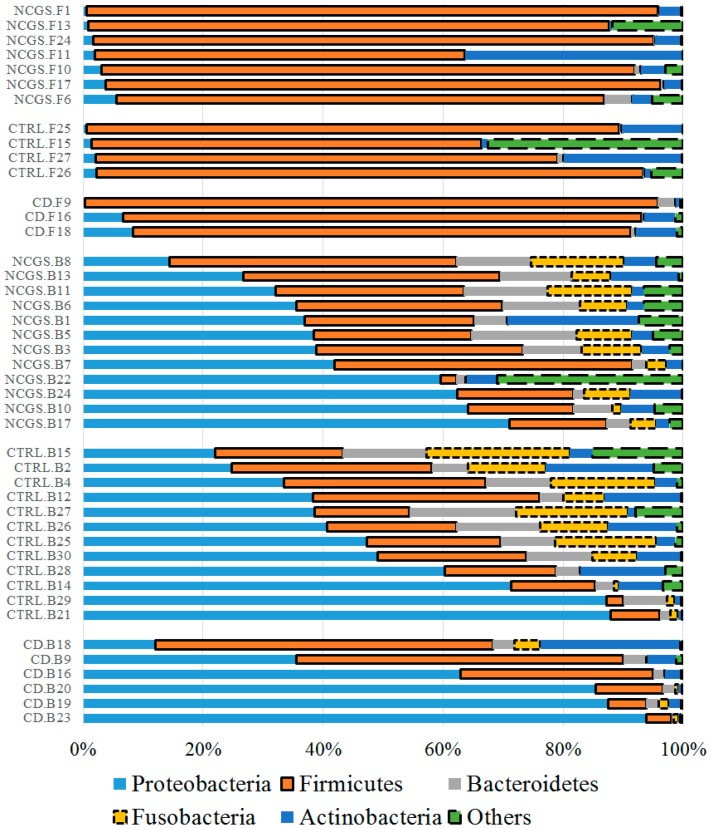
The taxonomic composition of duodenal (*n* = 30) and fecal (*n* = 14) microbiota at baseline at the phylum level. Please note that the samples were organized based on the highest abundant phylum for each subset of subjects. This figure was created using data from the full (i.e., unfiltered) OTU table, thus, allowing for a more complete taxonomic view of the samples. In the samples IDs: CD (celiac disease), NCGS (non-celiac gluten sensitivity), CTRL (control subjects), B: duodenal biopsy, F: fecal.

**Figure 2:**
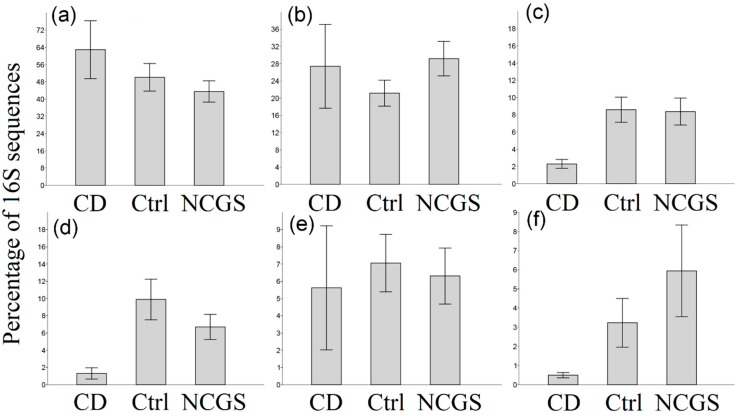
Bar charts showing the relative proportions of the 16S rDNA reads from the duodenal microbiota for all the main phyla. (**a**) Proteobacteria, (**b**) Firmicutes, (**c**) Bacteroidetes, (**d**) Fusobacteria, (**e**) Actinobacteria, (**f**) Others. Significant differences were only found in Bacteroidetes (**c**) and Fusobacteria (**d**), see main text. Bars represent the mean ± SE.

Overall 16S reads were assigned to a total of 27 phyla in all samples but only five phyla (Proteobacteria, Firmicutes, Actinobacteria, Bacteroidetes, and Fusobacteria) comprised the vast majority (>90%) of reads in most samples (Figure 1), as shown elsewhere. At the phylum level, there was a significantly lower abundance of Bacteroidetes (*p* = 0.022, Kruskal–Wallis test) and Fusobacteria (*p* = 0.052) in duodenal biopsies from CD patients (*n* = 30, Figure 2). This lower abundance of Bacteroidetes and Fusobacteria in CD patients was also true when analyzing the duodenal microbiota of women only (*n* = 22, *p* = 0.028 and *p* = 0.067, respectively).

**Figure 3:**
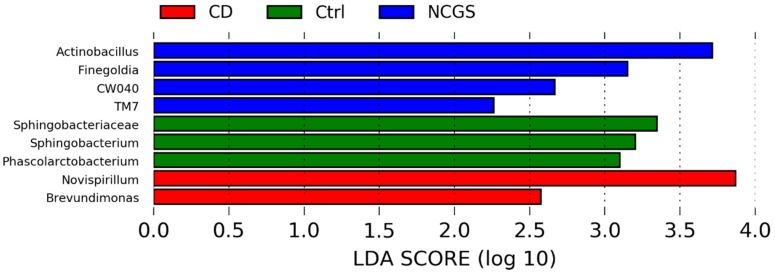
The LEfSe results from the comparison of the baseline duodenal microbiota. Please note that the bigger the LDA score, the bigger the contribution to the magnitude of the variation.

LEfSe analysis confirmed the finding of statistically significant differences in various bacterial groups among the three groups of subjects at the baseline (Figure 3). For instance, there was a higher abundance of *Actinobacillus* (Gammaproteobacteria), *Finegoldia* (Clostridia), and the phylum TM7 in NCGS patients, while *Sphingobacterium* (Bacteroidetes) was higher in the healthy subjects (Figure 3). The separate LEfSe analysis of samples from women confirmed the higher abundance of TM7 in NCGS patients and *Sphingobacterium* in healthy subjects and also revealed significant differences in various other bacterial groups (e.g., women with CD were deprived of Campylobacterales, Paraprevotellaceae, and Fusobacteriaceae; see Supplementary Figure S1). The health status of the patients was not related to significant differences in any index of richness or diversity with the exception of Shannon diversity index (lower in CD patients; Table 3 and Supplementary Table S1). This overall lack of difference in alpha diversity was also true when only analyzing samples from women (*n* = 22). 

**Figure 4:**
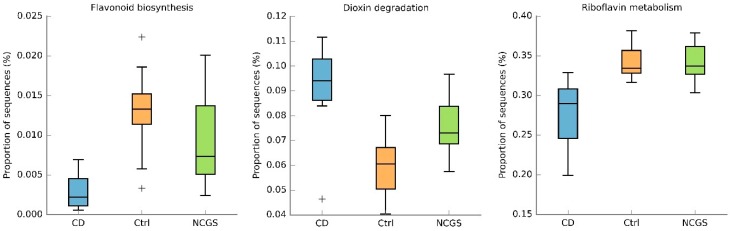
The box plots showing proportions of sequences for three metabolic features from the duodenal microbiota at the baseline. Plus (+) symbols represent outliers. Please note that there was no difference in any metabolic feature. CD: celiac disease, Ctrl: control, NCGS: non-celiac gluten sensitivity.

The differences in duodenal microbiota at the phylum (e.g., Bacteroidetes) and lower taxonomic levels (e.g., *Actinobacillus*) were not enough to differentiate the bacterial communities as a whole, as evaluated by the PCoA plots of weighted and unweighted UniFrac distances (Supplementary Figure S2) and this was also true when only analyzing the samples from women. The PICRUSt predicted metabolic features with the lowest uncorrected *p* values were flavonoid biosynthesis, dioxin degradation, and riboflavin metabolism (Figure 4) but after the Bonferroni correction, there was no significant difference in any metabolic feature. 

To summarize the results for the baseline duodenal microbiota, we found significant differences in the relative abundance of several bacterial groups but these differences were not enough to modify the diversity parameters (with the exception of Shannon diversity indexes) or predicted metabolic features.

#### 3.3.2. Microbiota in Fecal Samples at the Baseline

Only 14 samples were available for the analysis of fecal microbiota at the baseline. Fecal samples showed an unexpected high abundance of Firmicutes (~85%) and a low abundance of Bacteroidetes (~1%) regardless of the disease status (Figure 1). Despite the low number of samples, there was a clear higher abundance of fecal Ruminococcaceae in NCGS patients, and this difference was significant according to the LEfSe analysis (Supplementary Figure S3).

### 3.4. Effect of GFD on the Gut Microbiota 

#### 3.4.1. Effect of GFD on Duodenal Microbiota

All subjects had a GFD adherence above 90%. Sixty-seven percent of CD patients (4/6) and ninety percent of NCGS patients (9/10) reported a global improvement of symptoms after four weeks on GFD but this difference was not significant (*p* = 0.247, chi-squared test). There was also no difference in any other clinical or physiological parameter with the exception of abdominal pain (lower during GFD in CD patients, Table 4). 

**Figure 5:**
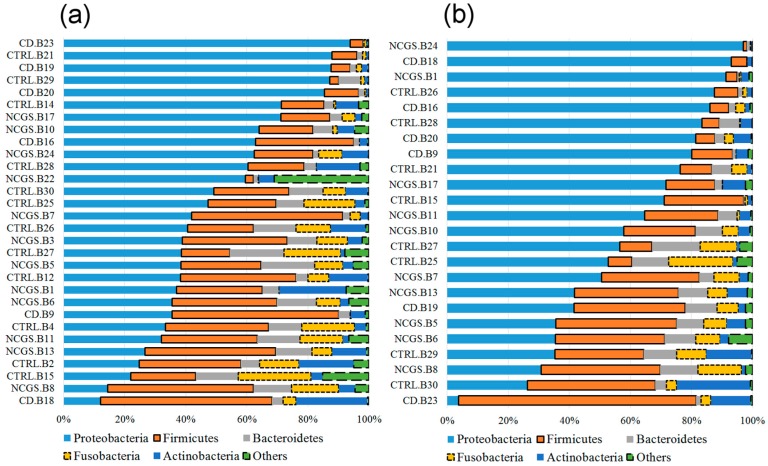
The taxonomic composition of duodenal microbiota between the baseline period ((**a**), *n* = 30) and the period after four weeks on GFD ((**b**), *n* = 24). Please note that the samples were organized based on the abundance of the most abundant phylum (i.e., Proteobacteria). In the samples IDs: CD (celiac disease), NCGS (non-celiac gluten sensitivity), CTRL (control subjects).

**Figure 6:**
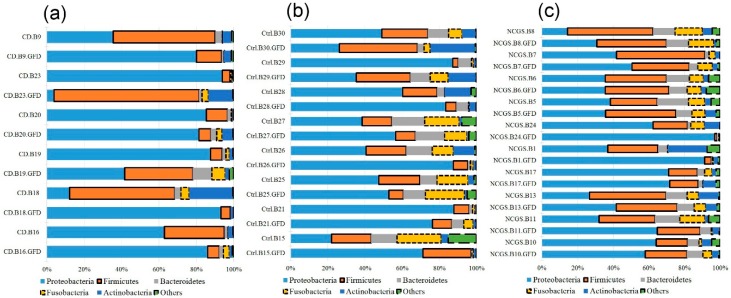
The taxonomic composition of duodenal microbiota for all paired samples from the CD patients (**a**), controls (**b**) and patients with NCGS (**c**). The purpose of this plot is to illustrate the over time variation within individuals. In samples IDs: CD (celiac disease), NCGS (non-celiac gluten sensitivity), Ctrl (control subjects).

**Figure 7:**
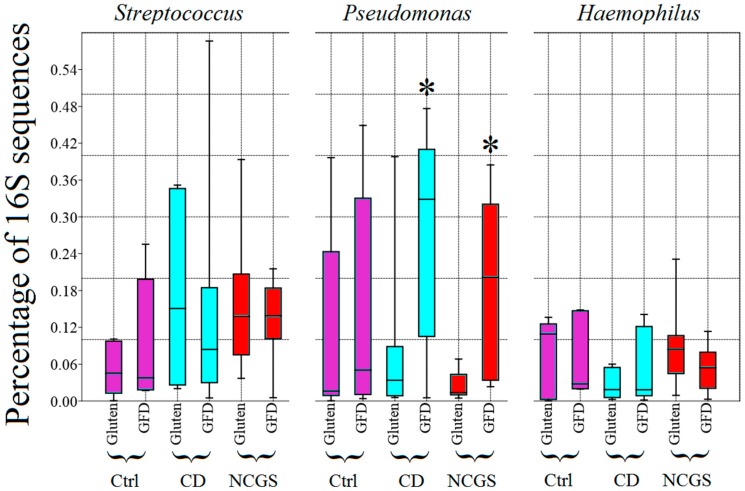
The box plots showing the relative abundance of 16S rDNA reads corresponding to the three most abundant bacterial groups in the duodenum at the genus level. * Significantly higher compared to the period of gluten consumption (*p* < 0.05, Wilcoxon signed-rank test). Please note that 90% (9 out of 10) and 50% (3 out of 6) of paired samples of patients with NCGS and CD (respectively) showed an increase in *Pseudomonas* (see main text for more details on this). CD: celiac disease, Ctrl: controls, NCGS: non-celiac gluten sensitivity, GFD: gluten-free diet.

An additional 2.9 million sequences (1.8 million from a total of 24 biopsy samples, and 1.1 million from a total of 12 fecal samples) were obtained from subjects on GFD. Paired samples were not obtained for all subjects mainly because of the lack of compliance, especially with the submission of stool samples (Table 1). Despite an apparently clear distinctive abundance and distribution of phyla in duodenum between the periods with and without dietary gluten (Figure 5), there was no significant difference in the abundance of any taxa between the two periods of time (*p* > 0.1), likely due to the high inter-individual variability. Additional analyses of relative abundances in paired samples revealed that each group of patients (controls, CD, and NCGS) displayed a distinctive variation over-time after consuming the GFD for four weeks (for example, most NCGS patients displayed little change after the GFD period, Figure 6). Interestingly, and despite a relatively more stable division-wide composition, 9 out of 10 paired samples of patients with NCGS showed an increase in the duodenal *Pseudomonas* on the GFD (Figure 7, baseline median: 1.3%, min/max: 0.47–6.8%; median after four weeks on GFD: 14.8%, 2.3–38.5%, *p* < 0.01, Wilcoxon signed-rank test, only subject 7 showed a decrease in this group, from 4.3% to 2.5%). This difference in most individuals was specific for *Pseudomonas* and not for other members of the duodenal microbiota (Figure 7). In contrast, only half of the paired samples (3 out of 6) from CD patients showed increases in *Pseudomonas* but these increases were so pronounced that they also affected median values (Figure 7). Additional analyses revealed that the 16S sequences from *Pseudomonas* were not different among the groups of subjects (see Figure S4 in “3.1 *Pseudomonas* in duodenum” in the Supplementary Information), thus suggesting that taxonomically similar *Pseudomonas* populations react differently in the presence of similar environmental conditions, in this case, in the absence of dietary gluten. This is particularly relevant in a context of the ecological significance of microdiversity [41]. 

**Figure 8:**
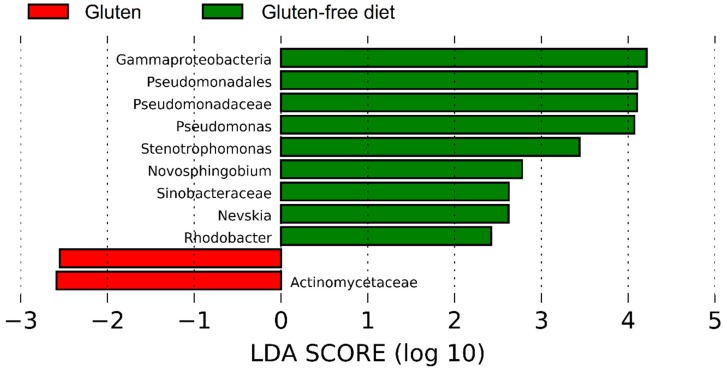
The LEfSe results of the duodenal microbiota from the comparison of the baseline and the period after four weeks on a GFD. These results are interesting because they also point out a potential difference in the *Pseudomonas* populations.

LEfSe analysis of the taxa at the genus level confirmed the results on *Pseudomonas* and showed that other Proteobacteria (e.g., *Stenophomonas* and *Novosphingobium*) were significantly more abundant on the GFD, while Actinomycetaceae was lower before the GFD (Figure 8). On the other hand, LEfSe did not reveal any taxa significantly associated with either a specific health status or with diet as class and health status as a subclass. Interestingly, LEfSe analysis revealed that *Brevundimonas* (a very low abundant group, <0.5% of all reads) was significantly enriched in CD patients when analyzing health status as class and diet as a subclass. This result was mainly due to a higher abundance of *Brevundimonas* in CD patients on the GFD. Other factors such as age, sex, or BMI were not significantly associated with the abundance of any bacterial taxa accordingly to the LEfSe analysis; however, this lack of significance must be taken cautiously because of the low sample size in each subgroup of patients. 

There was no significant difference in the bacterial richness or diversity in the duodenum when comparing the period at baseline and after four weeks on GFD or health status using either the full OTU table (Table 3) or the filtered OTU table (Table S1). The ANOSIM and Adonis tests revealed interesting results to the factors associated with the differences in microbial composition among the samples based on UniFrac distances (Table 5 and Supplementary Table S2). For example, the diet factor almost reached a level of significance when analyzing the weighted UniFrac distances (Table 5 and Table S2). Additionally, the grouping of duodenal samples based on health status was found to be statistically significant when using unweighted UniFrac distances and almost reached a level of significance when using weighted UniFrac distances (Adonis test, Table 5 and Table S2). The age of the patients also seemed to contribute to the separation of duodenal communities, especially when using the filtered OTU table (Table S2). These results were supported by significance in the ANOSIM test but the associated R values were very low (*R* < 0.10), indicating that the clustering of samples was relatively weak (Table 5 and Table S2). Please note that the analysis of both the full and the filtered OTU table revealed similar results, thus suggesting that low-abundant OTUs did not play an important role in the separation or lack thereof of communities. 

Considering the differences in relative abundance among the different bacterial groups of the duodenal microbiota (e.g., the higher *Pseudomonas* on GFD in CD and NCGS patients), we hypothesized that the beta diversity analyses for different bacterial populations may offer clues regarding the effect on different factors such as the diet and health status. Therefore, we used the Adonis and ANOSIM tests to compare UniFrac distances for either all non-Proteobacteria OTUs and *Pseudomonas* OTUs only (Supplementary Table S3). Despite obtaining low R values, thus suggesting the weak clustering of communities, this additional analysis revealed that the effect of these factors is different in distinct populations of microbes. For example, the effect of the BMI was stronger for *Pseudomonas* populations (Table S3).

#### 3.4.2. Effect of GFD on Fecal Microbiota

**Figure 9:**
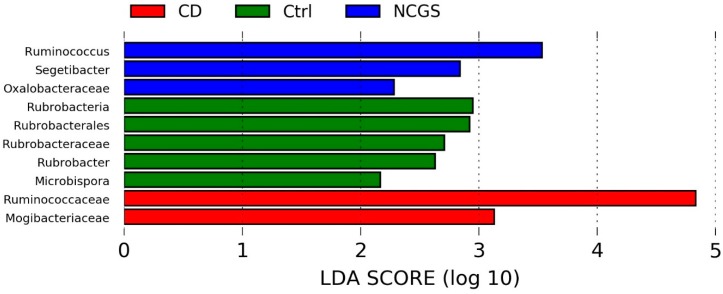
The LEfSe results of fecal microbiota from the comparison of all three groups of patients regardless of diet.

Gluten proteins are not completely digested in the small intestine and several members of the fecal microbiota have the capacity to metabolize gluten [42]; therefore, the removal of gluten from the diet may also affect the distal gut microbiota. In this study, however, both diet and health status were not associated with differences in fecal bacterial richness or diversity (Table 3 and Supplementary Table S1). LEfSe analysis did not find any indication to suggest a difference in fecal microbial communities according to diet as the class or diet as a class and health status as a subclass. Interestingly, the LEfSe approach revealed a diverse group of microorganisms that were significantly enriched in each of the disease states when using health status as the main class (Figure 9). The family Veillonellaceae, which was found to be lower in the feces of healthy subjects on GFD [21] and contains sulfite reducer members [43], was included in this group (higher in CD patients, Figure 9). The analysis of health status as class and diet as subclass revealed that Proteobacteria (in general, without an indication of a particular taxon within) was more abundant in CD patients. Beta-diversity analyses of UniFrac distances showed a significant grouping of fecal samples accordingly to BMIs and this relationship was also independent of low-abundant OTUs (Adonis test, Table 5 and Table S2).

#### 3.4.3. Effect of GFD on the Predicted Functional Profile

The closed OTU picking approach yielded a total of 4958 OTUs in biopsies and fecal samples. PICRUSt revealed no significant difference in the predicted functional profile of duodenal or fecal microbiota accordingly to diet or health status. Interesting results were found (for fecal samples only) when analyzing the group factor (six groups, control, CD and NCGS patients before and on GFD). For example, the proportions of genes related to the propanoate metabolism were higher in CD patients on a GFD (see “3.2 Predicted functional profile” in Supplementary Information) but caution must be exerted because of the low sample size in each subgroup of patients. 

## 4. Discussion

Increasing evidence suggests a role of the gut microbiota in the onset and clinical development of GRDs but this phenomenon has been mostly studied in Europe. This study sheds light for the first time into the complex host-microbiota interactions in control subjects and patients with CD and NCGS from México. Additionally, this study offers relevant clues regarding the potential effect of GFD on health and gut microbiota. 

The gluten metabolism is an interesting physiological phenomenon and growing evidence suggests a strong involvement of the gut microbiota [44]. However, each individual carries a highly specific group of microorganisms even at the strain level [1], and therefore such an involvement must be highly individualized. More importantly, the response of these unique communities to environmental factors (e.g., dietary changes, antibiotic administration) is also unique and may never return to the exact same baseline state before the challenge [45]. Finally, the region where the individuals live is an important factor, in fact, one study showed important interactions between the patients’ geographical location and the clinical and microbiological manifestations of inflammatory bowel disease [16]. In this study, for example, our results are unlikely to apply to patients with GRDs from other cities, even within the same state of Veracruz. 

From a clinical perspective, four weeks on GFD often improves symptoms and the quality of life in patients with CD or NCGS and this paper shows that this period of time was also enough to change the gut microbiota in our group of subjects, for example, duodenal *Pseudomonas* in NCGS patients. In contrast, Tjellström et al. [46] showed that fecal short-chain fatty acids output (a direct result of microbial activity) in CD patients with more than one year on GFD was significantly different compared to the output in CD patients with less than one year on GFD and CD patients at the presentation, thus suggesting that a long period of time on GFD may be necessary to fully re-establish the functioning of the gut microbial ecosystem in some patients. It has also been shown that a subgroup of patients does not respond positively even while adhering to a strict GFD and that these patients seem to harbor distinctive microbiota [47]. Here we showed that each individual carries a highly specific gut microbial composition, that the microbiota is different between healthy subjects and people with GRDs, and that this microbiota can experience variation due to the removal of gluten. It is important to note that this change also varies widely among individuals (the most significant and consistent change was associated with duodenal *Pseudomonas* in NCGS patients but every individual showed a unique increase or decrease in the abundance of these and other microorganisms). 

The (unexpected) finding of higher abundance of *Pseudomonas* in some patients during GFD deserves special attention. For instance, whether the increase in *Pseudomonas* is beneficial or not to the integrity of the duodenal mucosa is uncertain. Clinicians often associate *Pseudomonas* with diseases because of the pathogenic nature of some strains of *P. aeruginosa* and other species. However, *Pseudomonas* is a highly heterogenic bacterial genus that includes thousands of non-pathogenic, highly divergent strains inhabiting a wide variety of environments [48]. Unfortunately, very few studies have paid attention to native gut-associated *Pseudomonas* [49,50,51,52]. The finding that a GFD is associated with a higher abundance of *Pseudomonas* in the duodenum could be explained using at least two hypotheses. First, gluten may lead to a given immunological status in the mucosa that interferes negatively with the presence of autochthonous *Pseudomonas*, thus explaining the lower abundance at baseline. Second, some members of *Pseudomonas* may act as a protective microbe and its low abundance may prompt a more sensitive state to dietary allergens. This is supported by the relatively lower abundance of *Pseudomonas* in CD and NCGS patients before the GFD (Figure 7).

The possibility that some members of *Pseudomonas* can act as protective agents suggest that some strains of *Pseudomonas* may even be considered as probiotics for patients with some GRDs. Interestingly, Gao et al. [53] showed that *Pseudomonas* and other bacteria were reduced in cancerous tissues compared to adjacent non-cancerous tissues, thus suggesting a protective role in the gut mucosa. Wei et al. [54] identified an interesting aciduric gluten-degrading enzyme from *P. aeruginosa* with a therapeutic potential for CD; yet this does not explain whether GFD would lead to a higher or lower abundance of gluten-degrading *Pseudomonas* (we reasoned that gluten-degrading *Pseudomonas* populations would grow preferentially only if gluten consumption offers a selective advantage). One study showed higher abundances of *Pseudomonas* in the duodenum of adult CD patients on a GFD compared to controls but this finding was not discussed at all [55]. This current study also suggests that other non-*Pseudomonas* Proteobacteria (e.g., *Stenophomonas*) deserve attention in terms of gluten degradation and gut health.

This study also shows that the health status in terms of gluten sensitivity may be related to differences in the distal digestive microbiota. For example, this study showed a higher abundance of Ruminococcaceae in the fecal microbiota of NCGS patients. Additionally, Veillonellaceae, a pro-inflammatory taxon that has been shown to be increased in patients with inflammatory bowel disease and inflammatory bowel syndrome [56,57,58], was shown to be increased in fecal samples from CD patients. This adds valuable information to a growing literature showing that the distal microbiota is also worth looking at in gluten-related disorders [59].

This study has limitations that are relevant to future studies. First, this and other studies lack a large enough sample size to generalize phenomena and even with bigger samples sizes the results cannot be extrapolated from one population to others [17]. Second, gluten-free diets vary widely around the world and these may or may not lead to a microbial state more similar to healthy controls [13]. Third, 16S sequencing does not inform about the microbe-immune system interaction at the cell level. In this regard, De Palma et al. [60] showed interesting differences in IgA-coated fecal bacteria in treated and untreated CD patients, thus suggesting that a simple molecular characterization of microbes is not enough to fully capture the complex relationship. Fourth, one study showed that serum concentrations of short-chain fatty acids were similar in the control and CD patients; however, the authors found an interesting difference between genders [61]. This is particularly important because the reasons explaining the differences between genders with regards to the clinical presentation and severity of GRDs and other autoimmune disorders have not been fully clarified. One hypothesis suggests that infections can induce autoimmune diseases [62]. Finally, we only looked at the bacterial microbiota here, yet non-bacterial organisms (e.g., yeasts) may play a role in these disorders [63].

## 5. Conclusions

In summary, this study generates valuable preliminary data about the relationship between the gut microbiota and gluten-related disorders in Mexican people. Interestingly, the four-week consumption of GFD was associated with an increased abundance of *Pseudomonas* in duodenal biopsies of patients with these disorders, particularly in NCGS patients. This change was noticed despite a general lack of differences in richness or diversity. *Pseudomonas* comprises strains with gluten-degrading capabilities that deserves more attention. It is our hope that these results can contribute to starting to visualize alternatives for the more effective treatment of afflicted patients in our area. 

## Figures and Tables

**Table 1:** The health status, age (in years), BMI, sex, and sampling information for all our group of 30 subjects ^1^.

ID	Health Status	Age	BMI	Sex	Paired Duodenal Samples?	Paired Fecal Samples?
9	CD	35	27	Woman	Yes	Yes
16	CD	36	20	Woman	Yes	Yes
18	CD	62	18	Woman	Yes	Yes
19	CD	25	23	Woman	Yes	NA
20	CD	47	25	Woman	Yes	NA
23	CD	73	21	Woman	Yes	Only on GFD
1	NCGS	23	28	Woman	Yes	Yes
3	NCGS	21	24	Woman	Only baseline	NA
5	NCGS	24	25	Woman	Yes	Only on GFD
6	NCGS	23	29	Woman	Yes	Yes
7	NCGS	22	25	Woman	Yes	NA
8	NCGS	24	27	Woman	Yes	NA
10	NCGS	27	23	Man	Yes	Yes
11	NCGS	23	29	Man	Yes	Only baseline
13	NCGS	37	31	Woman	Yes	Yes
17	NCGS	59	19	Woman	Yes	Yes
22	NCGS	34	26	Woman	Only baseline	NA
24	NCGS	38	24	Woman	Yes	Only baseline
2	Control	23	33	Man	Only baseline	NA
4	Control	24	33	Man	Only baseline	NA
12	Control	23	24	Woman	Only baseline	NA
14	Control	25	23	Woman	Only baseline	NA
15	Control	26	21	Woman	Yes	Yes
21	Control	24	29	Man	Yes	NA
25	Control	45	28	Woman	Yes	Only baseline
26	Control	64	24	Man	Yes	Yes
27	Control	23	25	Man	Yes	Only baseline
28	Control	39	25	Man	Yes	NA
29	Control	58	26	Woman	Yes	NA
30	Control	42	27	Woman	Yes	NA

^1^ ID: patients’ internal identification number useful for retrieval of sequence information from the SRA (NCBI). CD: Celiac Disease; NCGS: non-celiac gluten sensitivity; GFD: gluten-free diet; BMI: body mass index. NA: not available for analysis.

**Table 2:** The baseline clinical, physiological, and other parameters among the groups of subjects ^1^.

	CD (*n* = 6)	NCGS (*n* = 12)	Controls (*n* = 12)	*p* Value
Proportion of women	100%	92%	50%	0.017
CD in family, %	67%	17%	8%	0.017
DQ2 or DQ8 positive, %	83%	50%	42%	0.217
Severe abdominal bloating (Likert), %	66%	81%	NA	0.121
Severe abdominal pain (Likert), %	50%	42%	NA	0.862
Age in years (median, range)	41.5 (25–73)	24 (21–59)	25.5 (23–64)	0.077
BMI, kg/m^2^, median (range)	21.8 (18–27)	25.3 (21–30)	25.2 (19–31)	0.050
Hemoglobin, g/dL, median (range)	12.3 (10.7–12.6)	13.8 (12.1–14.6)	13.8 (12.7–16)	0.050
Total cholesterol, mg/dL, median (range)	151 (110–222)	207 (116–323)	198 (136–299)	0.100
HDL, mg/dL, median (range)	38 (35–47)	43 (29–51)	36 (34–70)	0.013
LDL, mg/dL, median (range)	91.8 (63–161)	109 (75–143)	106 (79–186)	0.409
Triglycerides, mg/dL, median (range)	69.5 (40–230)	108 (62–270)	154 (83–277)	0.182
AST, median (range) UI/mL	29 (19–37)	23 (8–44)	26 (8–53)	0.523
ALT, median (range) UI/mL	22 (10–39)	19 (11–85)	24 (11–51)	0.895
Eosinophils DLP, median (range)	5 (0–22)	1.5 (0–13)	3.8 (0–11)	0.392
IEL in duodenum, median (range)	24 (15–39)	8 (0–22)	6 (0–12)	0.001

^1^*p* values come from the chi-squared test when comparing proportions (e.g., proportion of women) or the non-parametric Kruskal–Wallis test when comparing all other values. CD: Celiac disease; NCGS: non-celiac gluten sensitivity; DQ2 and DQ8 are haplotypes within the HLA-DQ serotyping system; BMI: body mass index; HDL: high density lipoprotein; LDL: low density lipoprotein; AST: aspartate aminotransferase; ALT: alanine aminotransferase; IEL: intraepithelial lymphocytes; DLP: duodenal lamina propria. NA: not applicable.

**Table 3:** The summary of the alpha-diversity indices from the analysis of all OTUs (full OTU table) from the duodenal microbiota accordingly to the diet and health status ^1^.

Biopsy Samples	Baseline (*n* = 30)	On GFD (*n* = 24)	*p* Value	Control (*n* = 20)	CD (*n* = 12)	NCGS (*n* = 22)	*p* Value
Richness	1127	1177	0.9562	1695	1687	1917	0.8450
PD whole tree	87	91	0.8961	1113	1057	1231	0.8426
Chao1	1746	1831	0.9924	88	86	91	0.9327
Shannon	5.5	5.5	0.8954	5.6 ^a^	4.8 ^a,b^	5.9 ^a,c^	0.0193
Fecal samples	Baseline (*n* = 14)	On GFD (*n* = 12)	*p* value	Control (*n* = 6)	CD (*n* = 7)	NCGS (*n* = 13)	*p* value
Richness	1692	1341	0.2519	1696	1347	1552	0.7946
PD whole tree	101	89	0.3217	99	86	98	0.6694
Chao1	2205	1826	0.2519	2192	1783	2088	0.8086
Shannon	5.5	5.2	0.7425	4.6	5.8	5.4	0.7551

^1^*p* values come from the Kruskal–Wallis test (same superscripts indicate lack of statistically significant difference). GFD: gluten-free diet; CD: celiac disease; NCGS: non-celiac gluten sensitivity. For clarity and lack of statistical significant difference for most values, only median values are presented.

**Table 4:** The clinical, physiological, and other parameters before and after four weeks of consumption of a gluten-free diet^1^.

	CD (*n* = 6)		NCGS (*n* = 12)		Controls (*n* = 12)	
	Baseline	On GFD	Baseline	On GFD	Baseline	On GFD
Hemoglobin, g/dL, median (range)	12.3 (10.7–12.6)	12.4 (12.2–13.3)	13.8 (12.1–14.6)	13.5 (11.6–12.10)	13.8 (12.7–16)	13.7 (12.7–14.5)
Total cholesterol, mg/dL, median (range)	151 (110–222)	160.5 (103–210)	207 (116–323)	185 (140–245)	198 (136–299)	175 (189–207)
HDL, mg/dL, median (range)	38 (35–47)	37.4 (35–37)	43 (29–51)	41 (14–53.5)	36 (34–70)	46 (15–65)
LDL, mg/dL, median (range)	91.8 (63–161)	104 (65–130)	109 (75–143)	121 (76–135)	106 (79–186)	120 (82–157)
Triglycerides, mg/dL, median (range)	69.5 (40–230)	118 (38–157)	108 (62–270)	93 (70–217)	154 (83–277)	194 (73–247)
AST, UI/mL, median (range)	29 (19–37)	21 (13–40)	23 (8–44)	20 (12–52)	26 (8–53)	19 (13–28)
ALT, UI/mL, median (range)	22 (10–39)	25 (16–38)	19 (11–85)	22 (9–V61)	24 (11–51)	26 (19–33)
Severe abdominal bloating (Likert), %	66%	0%	81%	25%	NA	NA
Severe abdominal pain (Likert), %	50%	0%	42%	14%	NA	NA
Eosinophils DLP, median (range)	5 (0–22)	10 (0–14)	1.5 (0–13)	1.5 (0–15)	3.8 (0–11)	4 (0–22)
Intraepithelial lymphocytes in duodenum, median (range)	12 (0–29)	11 (0–43)	0 (0–22)	7.5 (0–22)	5 (0–20)	7.5 (5–33)

^1^ The only parameter that showed statistically significant difference was severe abdominal pain (*p* < 0.05, chi-squared test). CD: Celiac disease; NCGS: non-celiac gluten sensitivity; GFD: gluten-free diet; HDL: high density lipoprotein; LDL: low density lipoprotein; AST: aspartate aminotransferase; ALT: alanine aminotransferase; DLP: duodenal lamina propria. NA: not applicable.

**Table 5:** The *R* and *p* values resulting from the Adonis and ANOSIM tests from the analysis of all OTUs (full OTU table) for each variable.

	**Biopsy Samples**		**Fecal Samples**	
**Adonis Test Results**	**Weighted**	**Unweighted**	**Weighted**	**Unweighted**
Diet	*p* = 0.053	*p* = 0.293	*p* = 0.406	*p* = 0.877
Disease	*p* = 0.072	*p* = 0.006	*p* = 0.323	*p* = 0.195
Group	*p* = 0.067	*p* = 0.080	*p* = 0.417	*p* = 0.494
Age	*p* = 0.119	*p* = 0.060	*p* = 0.299	*p* = 0.201
BMI	*p* = 0.401	*p* = 0.082	*p* = 0.007	*p* = 0.010
	**Biopsy Samples**		**Fecal Samples**	
**ANOSIM Test Results**	**Weighted**	**Unweighted**	**Weighted**	**Unweighted**
Diet	*R* = 0.038 *p* = 0.106	*R* = 0.014 *p* = 0.262	*R* = 0.009 *p* = 0.333	*R* = −0.071 *p* = 0.952
Disease	*R* = 0.078 *p* = 0.026	*R* = 0.058 *p* = 0.058	*R* = 0.082 *p* = 0.136	*R* = 0.055 *p* = 0.221
Group	*R* = 0.082 *p* = 0.039	*R* = 0.035 *p* = 0.174	*R* = 0.039 *p* = 0.307	*R* = −0.001 *p* = 0.482
Age	*R* = 0.083 *p* = 0.034	*R* = 0.081 *p* = 0.032	*R* = 0.009 *p* = 0.349	*R* = 0.009 *p* = 0.392
BMI	*R* = 0.034 *p* = 0.230	*R* = −0.050 *p* = 0.800	*R* = 0.132 *p* = 0.024	*R* = 0.155 *p* = 0.018

The variables included Diet (gluten/GFD), Disease (Control/CD/NCGS), Group (six groups of samples comprise this category: Control, CD and NCGS before and after four weeks of GFD), Age (Young/Old), BMI (high/low). *p* values that reached statistical significance (*p* < 0.05) or were close to reaching significance (*p* < 0.1) are highlighted in bold for better visualization.

## Supplementary Materials

The following are available online at http://www.mdpi.com/2072-6643/10/11/1641/s1, Detailed information about recruitment of participants, the diagnosis of CD and NCGS, as well as GFD intervention, Figure S1: LEfSe results from the separate analysis of baseline samples from women only, Figure S2: PCoA plots of unweighted and weighted UniFrac using the original OTU table and the filtered OTU table, Figure S3: relative abundance of fecal Ruminococcaceae at baseline, Figure S4: jackknifed tree based on weighted UniFrac distances, Table S1: summary of alpha-diversity indices from the analysis of the filtered OTU table accordingly to diet and health status, Table S2: R and *p* values resulting from Adonis and ANOSIM tests from the analysis of the filtered OTU table, Table S3: R and *p* values resulting from Adonis and ANOSIM tests from the analysis of the OTU table containing all non-Proteobacteria OTUs and *Pseudomonas* OTUs only in duodenal biopsies.
